# Value priorities and their relations with quality of life in the Baby Boomer generation of Lithuanian nurses: a cross-sectional survey

**DOI:** 10.1186/1472-6955-6-10

**Published:** 2007-11-08

**Authors:** Aurelija Blazeviciene, Irayda Jakusovaite

**Affiliations:** 1 Kaunas University of Medicine, Kaunas, Lithuania

## Abstract

**Background:**

The understanding of the values of nurses is especially important, since nurses constitute 80% of workforce in the healthcare system in Lithuania. In addition to that, nursing is one of the major constituents of healthcare. The aim of this study was to determine what values predominate in the cohort of Baby Boomer nurses, and to evaluate the relation of these values with quality of life using M. Rokeach's terminal and instrumental values scale. M.Rokeach distinguished terminal values (such as world peace, wisdom, and happiness), which are preferred end-states of existence, and instrumental values (such as responsibility and cooperation), which are preferred modes of conduct.

**Methods:**

We performed a representative anonymous questionnaire-based inquiry of nurses working in regional hospitals of Lithuania. The nurses who participated in the study were distributed into four work cohorts: the Veterans, the Baby Boomers, the Generation Xers, and the Generation Nexters. The majority of the nurses belonged to the Baby Boomers and the Generation Xers cohorts. Since in Lithuania, like in the whole Europe, the representatives of the Baby Boomers generation are predominating among working people, we selected this cohort (N = 387) for the analysis. The survey data was processed using the SPSS statistical software package

**Results:**

The main values in life were family security, tranquility, and a sense of accomplishment. However, such values as true friendship, equality, and pleasurable and leisured life were seen as rather insignificant. The most important instrumental values were honesty, skillfulness, and responsibility. Our study showed a statistically significant (albeit weak) correlation between the QOL and terminal values such as the sense of accomplishment, tranquility, equality, and pleasure, as well as the instrumental value – obedience. We detected a statistically significant relationship between good QOL and satisfaction with oneself, relationships with the surrounding people, and friends' support.

**Conclusion:**

The findings of our study showed that, although Lithuania was under a totalitarian regime for 50 years, both the terminal and the instrumental values of the Baby Boomers generation are very similar to those of the same generation in other countries.

## Background

The scientific study of human values has a long tradition in the fields of psychology and sociology. Values are conceived as guiding principles in life which transcend specific situations, may change over time, guide selection of behavior and events, and which are part of dynamic system with inherent contradictions [1]. The thinking about the nature of human values has been largely influenced by the work of Milton Rokeach. M. Rokeach defined the value concept as "an enduring belief that a specific mode of conduct or end-state of existence is personally or socially preferable to an opposite or converse mode of conduct or end-state existence"[2]. Rokeach distinguished terminal values (such as world peace, wisdom, and happiness), which are preferred end-states of existence, and instrumental values (such as responsibility and cooperation), which are preferred modes of conduct [3]. This distinction is important because it addresses two major questions in life: "What do I want to achieve?" and "How do I want to achieve it?" [4]. The understanding of the values of nurses is especially important, since nurses constitute 80% of workforce in the healthcare system. In addition to that, nursing is one of the major constituents of healthcare. Today a nurse is not merely an obedient performer of tasks assigned by the physician. A nurse works in the same team together with the physician and other healthcare professionals. In addition to that, when improving their practice skills, nurses acquire more self-confidence and ability to cooperate with physicians as equal work partners.

At present, researchers devote significant attention to questions about how cultural, economic, political and value systems influence the quality life We used our culturally learned values as a standard to determine whether we are as moral and competent as others, to guide our presentations to others, and to help us rationalize beliefs, attitudes, and behaviors that would otherwise be personality or socially unacceptable [5-7]. Quality of life is multi-dimensional and includes having, loving, being, and living in good health. Quality of life refers to the overall level of well-being of individuals. It indicates how well people fare in several dimensions of life, which are more or less consensually defined as reflecting important societal values and goals [8-10]. Some research suggests that there exists a relationship between human value orientations and the quality life [8,11,12]. Quality of life (QOL) means a good life and we believe that a good life is the same as living a life with a high quality. All great religions and philosophies have a notion of a good life ranging from saying that a good life is attained by practical codes of conduct to requests to engage in a certain positive attitude to life or to search into the depths of your own being. Notions about a good life are closely linked to the culture to which one belongs. When people in a Western culture view a good life, the cultural conditioning makes them tend to include happiness, fulfillment of needs, functioning in a social context, etc. [9,13]. Haas B. K. formulated a specific definition of the quality of life: "Quality of life is a multidimensional evaluation of an individual's current life circumstances in the context of the culture in which they live and the values they hold" [14]. Thus, the generalization of concepts presented by various authors leads to the statement that quality of life is primarily the subjective feeling of wellbeing, including the physical, the psychological, the social, and the spiritual levels.

Literature clearly shows that value-based attitudes and the evaluation of the quality of life depend on the person's age, i.e. to which generation he/she belongs [15]. A generation is defined as an identifiable group that shares years, age, location, and significant life events at critical development stages, divided by five to seven years into the first wave, the core group, and the last wave. A generational group, often referred to as a cohort, includes those who share historical or social life experiences, the effects of which are relatively stable over the course of their lives. These life experiences tend to distinguish one generation from another [16]. A cohort develops a personality that influences a person's feelings toward authority and organizations, what they desire from work, and how they plan to satisfy those desires [16]. Each generation has a unique perspective on the world of work. Its members tend to hold similar views about what is an attractive work environment; the nature of the team they would choose to be a part of; and – perhaps most confounding to instructors – preferences for acquiring, digesting, organizing, and distilling information and skills [17]. Understanding these generational differences is critical to instructors who try to advance the values, philosophy, knowledge and skills upon which the smooth running of the business depends. People of different perspectives always have the potential to bring different thoughts and ideas to problem solving and future opportunity. An unfortunate outcome, one that militates against positive creative synergy, is intergenerational conflict: differences in values and views, and ways of working, talking, and thinking that set people in opposition to one another and challenge the organization's best interests. Unfortunately, rebellion between groups that are different is an almost wired-in part of human nature. Not understanding others' perspective on the world can be stressful, confusing, and frustrating [16]. Different generations are united by socialization that allows individuals to engage in self-creation, to develop their possibilities, and – in case of need – to change [17]. Such changes became especially relevant after Lithuania became the member of the EU.

### The aim of the study

The aim of this study was to determine what values predominate in the cohort of Baby Boomer nurses, and to evaluate the association of these values with the quality of life.

### The objectives of the study

1. To evaluate what instrumental and terminal values predominate in the generation of Baby Boomer nurses.

2. To evaluate the associations of values with the subjective evaluation of the quality of life in the generation of Baby Boomer nurses.

## Methods

### Setting

In November 2006, we performed a representative anonymous questionnaire-based inquiry of nurses working in regional hospitals of Lithuania. We used the target sample in our study. In total, 1,000 questionnaires were distributed (N = 872; response rate – 87.2%). The nurses were given a questionnaire consisting of 12 terminal and 12 instrumental values listed according to M. Rokeach. The respondents were asked to rank each list of 12 values in order of their importance as guiding principles in their lives. Questions from the WHO questionnaire were used for the overall evaluation of the quality of life. The respondents were asked to rank their disagreement/agreement with the provided statements on a five-point Likert scale.

### Sampling

The nurses who participated in the study were distributed into four work cohorts: the Veterans, the Baby Boomers, the Generation Xers, and the Generation Nexters. The majority of the nurses belonged to the Baby Boomers and the Generation Xers cohorts. Since in Lithuania, like in the whole Europe, the representatives of the Baby Boomers generation are predominating among working people, we selected this cohort (N = 387) for the analysis. Baby Boomers are people born between 1943–1960, they were born during or after World War II, and were raised in the era of extreme optimism, opportunity, and progress. They are the nation's largest demographic group. The study met all ethical standards. Since our study had no clinical impact, verbal consent of the respondents was sufficient. The respondents were familiarized with the aim of the study and were informed that participation in the study is voluntary, and all the obtained data would be anonymous and used strictly for research purposes.

### Data analysis

The survey data was processed using the SPSS statistical software package (version 13). Rank mean values and ranks were used for the determination of value priorities. The reliability of the statistical data was verified according to the χ^2 ^test, degrees of freedom number (df), and statistical significance. The relationship between two independent variables was assessed by calculating ***Kendall***'s rank correlation coefficients, taking into consideration the value of the correlation ratio and its statistical significance (reliability notation: p < 0.05 means statistically significant, and p < 0.01 – highly significant).

## Results

### Priorities of terminal and instrumental values in the Baby Boomers generation

Personal values of nurses and physicians have not been studied much, although nurses' and physicians' values are crucial to health care practice, and have come under scrutiny during recent years, especially regarding priorities in health care. Despite the fact that research regarding personal values of nurses and physicians is sparse, values and their distribution among various types of population have been thoroughly investigated [18].

We analyzed ranked terminal and instrumental values. Terminal values are the goals in life that we think are most important and that we feel are most desirable. Instrumental values are basically the kind of personal characteristics that we think highly of [7]. As seen in the presented Table, according to the Baby Boomers generation, the main values in life were family security, tranquility, and a sense of accomplishment. However, such values as true friendship, equality, and pleasurable and leisured life were seen as rather insignificant. The most important instrumental values were honesty, skillfulness, and responsibility. Such values as obedience and joyfulness were considered to be absolutely unimportant (see Table 1.)

**Table 1:** Rankings and Composite Rank Orders of the Instrumental and Terminal Values for the Baby Boomers Generation of Nurses

**Terminal values**	**M**	**Rank order**	**Instrumental values**	**M**	**Rank order**
Comfortable life (a prosperous life)	4.59	4	Ambitious (hard-working, aspiring)	5.91	5
A sense of accomplishment (a lasting contribution)	4.38	3	Capable (competent, effective)	3.88	2
Tranquility	4.30	2	Cheerful (lighthearted, joyful)	7.74	10
A world beauty (beauty of nature and the arts)	7.32	8	Courageous (standing up for your beliefs)	5.85	4
Equality (brotherhood, equal opportunity for all)	7.72	10	Helpful (working for the welfare of others)	6.76	8
Family Security (taking care of loved ones)	2.69	1	Honest (sincere, truthful)	3.39	1
Freedom (independence, free choice)	5.99	6	Imaginative (daring, creative)	6.15	7
Inner harmony (freedom from inner conflict)	5.13	5	Logical (consistent, rational)	6.02	6
Pleasure (an enjoyable leisurely life)	8.65	11	Loving (affectionate, tender)	7.53	9
Social recognition (respect, admiration)	6.74	7	Obedient (dutiful, respectful)	8.16	11
True friendship (close companionship)	7.65	9	Responsible (dependable, reliable)	3.96	3

### Relation between values and the evaluation of the quality of life

This study investigated the relationships between individual values and quality life. The findings showed that more than one-half of the nurses of the Baby boomers generation positively evaluated their quality of life (52.8 %). They were also satisfied with their appearance (60.0%) and themselves in general (78.1%). Over two-thirds of the nurses were satisfied with their personal relationships with the others and with their friends' support. Nurses who positively evaluated their quality of life were also satisfied with their appearance, with themselves, and with their relationships with other people. Statistically significant differences were detected in this respect. Meanwhile, no statistically significant differences were found between nurses who were satisfied with their quality of life and those satisfied with their friends' support (see Table 2).

**Table 2:** Satisfaction with life among nurses of the Baby Boomers generation who were satisfied with their QOL

	%	χ^2^	df	p
Satisfied with their appearance	96.7	15.96	2	p < 0.05
Satisfied with themselves	97.2	36.4	2	
Satisfied with relationships with other people	82.6	17.5	2	
Satisfied with their friends' support	76.1	1.4	2	p > 0.05

* p < 0.05; ** p < 0.01

The majority of researchers analyze how values are related to the quality of life. In our study, we also analyzed the relations between values and the evaluation of the QOL. Our study showed a statistically significant (albeit weak) correlation between the QOL and terminal values such as the sense of accomplishment, tranquility, equality, and pleasure, as well as the instrumental value – obedience (see Table 3).

**Table 3:** Kendall's correlation coefficient between the nurses' terminal and instrumental values and their subjective evaluation of QOL

	Terminal and instrumental values				
	
	A sense of accomplishment	Tranquility	Equality	Pleasure	Obedience
Good quality of life	0.385**	0.082	0.060	0.062	0.086
Satisfaction with one's appearance	0.235**	0.096*	0.096**	0.074	0.081
Satisfaction with oneself	0.312**	0.068	0.065	0.102*	0.133**
Satisfaction with one's relationships with the surrounding people	0.198**	0.038	0.093	0.071	0.149**
Satisfaction with the friends' support	0.093	0.019	0.144**	0.119*	0.033

* p < 0.05; ** p < 0.01

Finally, we analyzed the inter-correlation between satisfaction with the quality of life and satisfaction with oneself, relationships with the surrounding people, and friends' support. We detected a statistically significant relationship between good QOL and satisfaction with oneself, relationships with the surrounding people, and friends' support. We also detected a correlation between satisfaction with oneself and satisfaction with relationships with the surrounding people, and friends' support (see Table 4).

**Table 4:** Kendall's correlation coefficient of QOL

	1	2	3	4
Good quality of life (1)	1.00			
Satisfaction with oneself (2)	0.269**	1.00		
Satisfaction with relationships with the surrounding people (3)	0.146**	0.318**	1.00	
Satisfaction with the friends' support (4)	0.084*	0.188**	0.370**	1.00

* p < 0.05; ** p < 0.01

## Discussion

Understanding of values requires us to understand their relationship with needs. Already in early 60s, Western countries noticed a new tendency of changes in cultural values, manifesting itself through the populations' decreasing interest in their material welfare and physical security, and increasing attention paid to the non-material (or post-material) values, such as freedom, self-expression, individualism, tolerance, and participation in important social decision-making [6]. At present, different value-based attitudes in different generations are widely discussed. Baby Boomers value individuality and youth and are self-absorbed. Baby Boomers were born between 1943 and 1964. The overall characteristic of Baby Boomers leads to the conclusion that their core values are optimism, team orientation, personal gratification, health and wellness, personal growth, youth, work, and involvement. Baby Boomers also redefined roles, promoted equality, left unfulfilling relationships to seek more fulfilling ones, sought immediate gratification, and manipulated the rules to meet their own needs. At work, Baby Boomers are service-oriented, driven, willing to go the extra mile, good at relationships, want to please, and are good team players. They are not naturally "budget minded", uncomfortable with conflict, reluctant to go against peers, may put process ahead of pursuit, are overly sensitive to feedback, are judgmental of those who see things differently, and are self-centered [15].

Lithuania has been undergoing tremendous transformation in the sphere of politics, economy and culture. The consequences of this transformation are still taking shape, and elements of the older culture are still widespread, but can any major features of new pattern be discerned? In the process of European integration, the problem of value change remains very significant if we are to understand processes of change not only in the economy, politics, and society in general, but in minds, outlooks an social behavior of people as well [19,20]. During 16 years of the Independence, there has been an on obvious transition in the scale of values from the socialist principle of equality (the next-to-last position) towards the principle of individualism. A similar situation developed with respect to "true friendship" – it dropped to the 9^th ^position. The principle of collectivism is dropping out of favor. Although Lithuania has been undergoing political and economic changes for only 15 years, but the values of the nurses of the Baby Boomers generation in Lithuania are now similar to those of the same generation in other countries. For instance, the results of the study performed by Western researchers were similar to those obtained in our study. In their study, the most important terminal values were found to be internal harmony and tranquility, whereas the least important ones were beauty and salvation. The most important instrumental values were honesty and love, whereas the least important ones were politeness and obedience [21,22]. The study performed by Canadian scientists showed that physicians of the Baby Boomers generation were less devoted to a physician's career, compared to the Generation X (Xers) physicians [23]. These results closely resembled those obtained in our study, since our respondents pushed the value "social recognition" down to the 7^th ^position. The findings of the study performed by Finnish researchers, where values of physicians were analyzed, showed that the most important values for the studied population were family, health, and friendship [17]. These results also closely resemble those obtained in our study.

The value-based attitudes of an individual are very closely related to his/her attitude to the quality of life. People who positively evaluate their quality of life and are satisfied with their lives are probably more tolerant and possibly also more creative and assertive [24]. Individuals who give priority to the materialistic approach present more negative evaluations of their QOL. Materialism directly correlates with depressive and neurotic mood and behavior [25]. Good life has a number of prerequisites – financial wellbeing, health, education, social integration, etc. Although one cannot deny the importance and the influence of the objective economic and social factors on the quality of life, yet the most important factors are an individual's ability to form his/her life with respect to his/her needs, and the ability to fulfill his/her aims.

## Conclusion

In this paper, we empirically investigated terminal and instrumental values of nurses from the Baby Boomers generation. The findings of our study showed that, although Lithuania was under a totalitarian regime for 50 years, both the terminal and the instrumental values of the Baby Boomers generation are very similar to those of the same generation in other countries. During 16 years of the Independence, there has been an on obvious transition in the scale of values from the socialist principle of equality and collectivism towards the principle of individualism. In this context, it is not so important to have outward control over a person – rather, it is much more important to organize circumstances and situations in such a way that it would be possible and useful for a person to do what has to be done for the society. The positive evaluation of the quality of life statistically significantly positively correlated with the most important terminal values such as "the sense of accomplishment" and "tranquility". Although such values as "equality" and "pleasure" were considered to be unimportant, they also statistically reliably positively correlated with positive evaluations of QOL.

## Competing interests

The author(s) declare that they have no competing interests.

## Authors' contributions

AB and IJ contributed equally to the writing of this paper.

## Pre-publication history

The pre-publication history for this paper can be accessed here:


